# Evaluating image modification as a harm reduction approach in content moderation

**DOI:** 10.1007/s00426-026-02241-5

**Published:** 2026-03-26

**Authors:** Sarah Lewitzka, Alana Liebeknecht, Victoria M. E. Bridgland, Ella K. Moeck, Reginald D. V. Nixon, Carolyn Semmler, Melanie K.T. Takarangi

**Affiliations:** 1 Flinders Institute for Mental Health and Wellbeing, Flinders University, Adelaide, South Australia Australia; 2 School of Psychology, University of Adelaide, Adelaide, Australia

## Abstract

Content moderators are repeatedly exposed to disturbing material, leaving them susceptible to harm, including post-traumatic stress disorder (PTSD) symptoms. Currently, social media platforms such as Facebook allow moderators to modify (i.e., blur or greyscale) the visual features of images to mitigate the potential impact of the material. However, there is little empirical basis for this intervention. Therefore, we examined whether viewing disturbing images in blur or greyscale, compared to viewing unmodified images, would influence mock content moderators’ anxiety, affect, and image-related intrusions. In Study 1, we randomly allocated participants (*n* = 309) to view either greyscaled, blurred, or unmodified images within a simulated content moderation task. In Study 2, participants (*n* = 365) chose the format of images, aligning with the choice real world content moderators have. Participants in both studies recorded and rated problematic characteristics of image-related intrusions they experienced and completed pre- and post-task measures of state anxiety and positive and negative affect. Blurring and greyscaling did not reduce any negative effects of viewing disturbing images, even when participants *chose* to view blurred or greyscaled images (which was only 14% and 16% of the time, respectively). Participants may have imagined the absent visual features of blurred or greyscaled images, such that they still visualized these images as if they were unmodified. Our data indicate that fixed blurring and greyscaling, under the conditions tested, did not mitigate immediate adverse outcomes, highlighting the need for further research to ensure moderator protection strategies are grounded in robust empirical evidence.

**Supplementary Information:**

The online version contains supplementary material available at 10.1007/s00426-026-02241-5.

## Introduction

As social media use continues to grow, so too does the need for content moderation. Billions of images and videos are uploaded daily to online platforms like Facebook and X, and a subset of this content—including beheadings, animal cruelty, and child abuse—is assessed and actioned by content moderators. Viewing streams of traumatic content has led to moderators reporting anxiety, depression, and posttraumatic stress disorder (PTSD) symptoms (i.e., intrusive memories or “intrusions” of content, avoiding reminders of content, and negative changes in their cognitive and emotional state) due to their work (Newton, 2019a; Spence et al., 2023a, b). One intervention that companies like Facebook have implemented to mitigate this negative impact is image modification: moderators can choose to blur or greyscale images (Meta, 2024). However, limited research speaks to the efficacy of image modification. Therefore, here we aimed to evaluate whether image modification influences people’s image-related intrusions, mood, and anxiety.

Although artificial intelligence programs can identify and remove most content that violates guidelines, human content moderators are required to assess approximately 700-2,000 more nuanced violations (e.g., torture) per day (Gillespie, 2020). Hence, it is essential to find effective ways to mitigate psychological harm for human moderators. One feasible way to reduce harm is by modifying the content itself—for example, by blurring or greyscaling images. Indeed, Facebook currently offers this strategy to their moderators (Meta, 2024). However, Meta provides little detail on how or when this strategy is implemented in practice, and a recent survey of content moderators revealed that access to this strategy varies (Takarangi et al., 2026). Further, anecdotally, content moderators perceive these tools as inhibiting their ability to conduct work accurately (Spence et al., 2023a, b). But regardless of accuracy, *when used*, does image modification reduce harm?

## Evidence for image modification

There are reasons to expect image modification may reduce the negative effects of moderating content. First, we know that colors can increase people’s attention and emotional arousal in response to stimuli, contributing to subsequent memory enhancement (for a review, see Dzulkifli & Mustafar, 2013). For example, red is associated with negative emotions (Sutton & Altarriba, 2016), negative perceptions of stimuli (Buechner & Maier, 2016), and self-reported emotional arousal (e.g., Jeong et al., 2011). Further, shifting images from color to greyscale decreases activation in the limbic system, the brain area responsible for emotional arousal (Codispoti et al., 2012). Thus, we would expect that people who view greyscaled (vs. color) images to be less negatively affected by those images. Second, *blurring* out details of disturbing images might also reduce their emotional impact by reducing the clarity/visibility of emotionally salient content (e.g., injuries, weapons, facial expressions). There is some empirical evidence that we review below, albeit limited and mixed, to support these ideas.

In one example, outside the content moderation context, Besançon et al. (2020) examined the effect of color manipulations (e.g., blue hue filter) and artistic stylizations (e.g., watercolor filters) for reducing repulsiveness of graphic surgical content. The researchers expected image modifications to reduce repulsiveness by obscuring the details (e.g., gory wounds or disgusting bodily fluids) that make viewing the image an emotionally negative experience, thereby ‘muting’ negative emotions and promoting neutral emotional responses (Mould et al., 2012). As expected, most modification techniques reduced how repulsive participants perceived the images to be, compared to unmodified images. However, the within-subjects design might have induced demand effects; seeing both versions may have led participants to infer that they were expected to rate modified images as less repulsive than the unmodified ones.

In a second example, Das et al. (2020) tested six groups of mock content moderators: a control group who viewed unblurred images, three groups who could adjust the blur (e.g., via a click, hovering over parts of the image, or using a slider tool to adjust the level of blur), and two groups who viewed the images with a fixed blur. Participants in the control group reported significantly lower positive affect—measured with the Positive Affect Negative Affect Scale (PANAS; Watson et al., 1988)—immediately following the content moderation task than the groups who had the choice to change the level of blur with the slider tool, but similar positive affect to the fixed blur group. These results suggest that having control over the blurring tool increased positive affect, perhaps because it increased participants’ sense of agency, or because participants expected that their chosen level of blur would improve their experience.

Third, Karunakaran and Ramakrishan (2019) looked at both greyscaling *and* blurring (separately). Both interventions included real content moderators who reviewed unmodified images for two weeks, before reviewing either greyscale images (*n* = 76) or blurred images (*n* = 37) for two weeks. Participants reported positive and negative affect using the PANAS at the end of the two-week unmodified period, and again at the end of the two-week intervention period (i.e., greyscale or blurred images). The greyscale intervention *increased* moderators’ positive affect whereas the blur intervention *decreased* positive affect, and neither intervention influenced negative affect. In their feedback on the interventions, moderators suggested that greyscaling lessened the emotional impact of the images, while the blur was frustrating to work with, perhaps explaining the differences in positive affect. Notably, this study likely did not have adequate statistical power to detect smaller effects of the intervention that may have been present for negative affect.

Taken together, the mixed evidence on image modification raises the alternate possibility that, while mitigating some negative outcomes, greyscaling and blurring might simultaneously create others. Perhaps when color or clarity is reduced, people imagine those missing elements in greater detail. For example, someone might see a blurred image of a person on the ground and imagine a dead body, even though it is actually a person sleeping. Similarly, if they see a black-and-white image of a spilled liquid, they might assume it is a puddle of blood. This process could intensify the emotional impact of the content: imagined events are sometimes perceived as having occurred in reality (imagination inflation; Garry et al., 1996), meaning imagined details may feel sufficiently real to evoke emotional reactions akin to those produced by direct exposure. Additionally, by using imagination to fill in gaps, people create a network of associations that make the memory more accessible—including involuntarily—in a variety of contexts (Tulving, 1972), especially when exposed to relevant reminders (e.g., dead bodies or blood). In summary, greyscaling or blurring content could have paradoxical effects: while potentially reducing the immediate emotional impact of an image, these techniques may inadvertently lead to enhanced imagination—especially when imagining blurred content or missing content, which is likely more effortful—and in turn greater emotional impact, and memory accessibility.

## The current study

We investigated whether image modification strategies (blurring and greyscaling) influence negative outcomes. In Study 1, we randomly allocated participants to view either greyscaled, blurred, or unmodified images within the content moderator simulation (a task we developed that simulates a real content moderators’ role and consistently produces increases in negative affect and state anxiety; Lewitzka et al., 2025). In Study 2, participants chose the format of the images (akin to real content moderators choosing whether to blur or greyscale the content they view). While Study 1 maintained experimental control with random allocation to conditions, Study 2 achieved ecological validity by mimicking content moderators’ autonomy to select their preferred viewing format. In both experiments, we measured participants’ positive affect, negative affect, and state anxiety levels before (pre-task) and after viewing the images (post-task). Unlike the previous research, we also measured both the frequency of intrusions and their associated problematic characteristics because intrusions are a frequently occurring symptom (e.g., Isvoranu et al., 2021) implicated in the development and maintenance of PTSD (Bryant et al., 2017) and known to both activate and exacerbate other PTSD symptoms (such as avoidance and negative mood; Haag et al., 2017) and deteriorate global mental health (Singh et al., 2020). Further, frequent intrusions contribute to ongoing distress (Craig et al., 2014), and the distress associated with intrusions—along with characteristics such as vividness—maintains and perpetuates intrusions over time (e.g., Marks et al., 2018). As such, interventions that reduce people’s emotional reactions to content should, in turn, make intrusions less likely to occur and cause distress (Ripley et al., 2017).

Across both studies, we expected that, if image modification works as intended, participants who viewed either the blurred or greyscaled images would have a smaller decrease in positive affect, and a smaller increase in negative affect and anxiety from pre- to post-task, and less frequent and problematic intrusions, compared to participants who viewed unmodified images. We also expected to find larger effects for the greyscale versus unmodified image conditions compared to the blurred versus unmodified conditions. We anticipated that the difference between the modified and unmodified groups would be more pronounced in Study 2, given that *choosing* to view modified images may result in expectancy effects (e.g., believing that the images will have less impact when less detail is visible, and therefore being less impacted by the images).

## Transparency and openness

The Flinders University Ethics Committee approved these studies (ID: 5653) and the studies were conducted in compliance with APA ethical standards. We report how we determined our sample size, all data exclusions, all manipulations, and all measures for each study. We pre-registered the studies prior to data collection (Study 1: https://osf.io/6ytkj/overview; Study 2: https://osf.io/thpja/overview) and the data for each study are publicly available (Study 1: https://osf.io/3dq6h/overview; Study 2: https://osf.io/9gake/overview), along with the variable codebook and all supplementary files (https://osf.io/ck6ab/overview). We used SPSS (Version 29.0.2.0) and JAMOVI (Version 2.4.12) for data analyses.

## Statistical overview

We assessed all data with a Q-Q plot for normality and with a boxplot for outliers. Data across both studies were normally distributed except for the intrusion frequency measure (positively skewed). There were outliers on multiple measures (i.e., negative affect change, positive affect change, state anxiety change, intrusion unwantedness, intrusion intent, PCL), however, when we removed these outliers, the pattern of results did not change, so we report our analyses on the full datasets here. There was homogeneity of variances for most measures. Where homogeneity of variances was violated (e.g., on some intrusion characteristic items and exploratory items), results from Welch ANOVAs and t-tests are reported instead of standard one–way ANOVAs and t-tests. Where there were no differences between groups, we quantified the level of evidence for the null hypothesis using Bayes Factors (BF10) as outlined by Wetzels et al. (2011)^1^. Although we used Bayesian analyses this way in both studies, we only pre-registered using it for Study 2. We used a default prior in our analyses, given the absence of strong theoretical or empirical grounds to specify an informed prior. We include Bayes Factors here to supplement our frequentist results and to provide an additional perspective on the strength of evidence, while acknowledging the limit of their interpretive value in the absence of strong prior information. All supplementary analyses are available in Online Resource 1.

## Study 1

### Method

#### Participants

We recruited all participants, including those used for pilot testing, from Amazon’s Mechanical Turk (MTurk), a crowd-sourcing method that allows quick and effective data collection from diverse samples (Shapiro et al., 2013). Based on an a priori power analysis in G*Power (Faul et al., 2009), 303 participants (101 per condition) were required for a 3-groups one-way ANOVA to detect a small-medium effect (*f* = 0.18; the smallest effect of interest we could target with available resources) at 80% power and α = 0.05. Therefore, we aimed to collect 303 participants for the main study.

We collected data from 369 participants in 2023 who were over 18 and had color vision, per our inclusion criteria. Participants did not require content moderation experience to participate. In line with our pre-registration, we excluded data from 40 participants who provided an incorrect answer to one or more of the eight neutral or ambiguous images (see *Image Stimuli* for explanation) that did not violate any ‘guidelines’. Similarly, we excluded data from 15 participants who provided an incorrect answer to more than three of the 14 images clearly containing violating content. We excluded data from an additional two participants who failed both of our embedded attention checks, two participants who experienced technical difficulties during the intrusion monitoring task, and one participant who guessed the main hypothesis. Our final sample of 309 participants ranged from 20 to 80 years of age (*M* = 43) and were mostly women (57.0%) or men (41.4%), with the remainder of the sample indicating they were non-binary or that they preferred not to say. Twenty-four participants (7.8%) indicated that they had experience as a content moderator, but some participants may have interpreted content moderation more broadly than our intended focus on reviewing graphic or disturbing visual content (i.e., referring to experience of other moderation such as spam or text-based material).

#### Materials

We conducted this study using Qualtrics web-based survey software (Qualtrics Software, 2018). All instructions and information we provided participants are available in Online Resource 2.

#### Image stimuli

We selected 24 negative images that elicited feelings of moral and/or physical disgust, based on ratings from pilot participants (*n* = 200). We asked pilot participants (*n* = 122 across several versions) to judge whether images violated any guidelines (and if yes, select which) and examined the “consensus” judgments. On the basis of these data, we classified 14 of the negative images—which had high consensus (> 93% “yes” responses) across different image formats—as objectively violating “community standards,” simulating the type of content that moderators would make judgments about in a real-life setting. These types of content are described in Facebook’s “community standards” – a set of guidelines outlining what is prohibited on Facebook (see: https://transparency.fb.com/en-gb/policies/community-standards/), including dead bodies, self-harm, torture, animal abuse, hate speech, and vandalism. For the other 10 negative images, it was more subjective whether these images explicitly violated any of the guidelines (consensus ~ 35–89%). For example, an image of a knife held to someone’s neck without visible injury could be seen as violating guidelines for depicting torture by one person, while another might judge it as a non-violation due to the absence of wounds or blood. We selected images where consensus ratings were as similar as possible across image format.

There were eight other images in the set (32 images in total). Five were ambiguous in nature; that is, they have both a neutral and a potentially negative interpretation, but they ultimately do not violate any guidelines and are not graphic or disturbing (e.g., a soldier being carried on a stretcher, but no injuries are visible). We included subjective and ambiguous content to ensure the study’s procedure reflected a real content moderation role. In a separate study, we asked real content moderators (*n* = 14) to review our simulation and they indicated that the simulation accurately reflects their role. The other three images were neutral (e.g., park scenery) and served to check participants were responding appropriately. The 32 images shown to the three groups were identical, the only difference was their format: greyscaled, blurred, or unmodified.

In developing the set of blurred images, we aimed to blur the images enough to obfuscate disturbing details, while keeping images discernible enough for participants to assess whether the content violated guidelines. To determine the appropriate range of blur, we pilot tested images with varying degrees of Gaussian blur, asking participants (*n* = 22) to describe the images to gauge whether they were able to discern the image’s content accurately. We exchanged any images that participants could not identify correctly or altered the level of blur. Images in the final blurred set ranged from 10 to 20% Gaussian blur.

#### Content moderator training and simulation task

We first told participants about content moderation and provided them with instructions for the task. Next, we showed them a set of guidelines modelled from the section of Facebook’s community standards that pertains to the types of imagery not permitted on their platform. We tested participants on their understanding of these guidelines with a set of practice questions. Participants then viewed the images and identified whether each image violated Facebook community standards (*yes/no*). If they answered yes, they selected which policy was violated: *(a)* a dead body in a non-medical setting, *(b)* self-injury, *(c)* hate speech, *(d)* harm against an animal, *(e)* harm against property, *(f)* torture, *(g)* violent death. We used this decision procedure for the simulation because Facebook claims that this procedure yields the most accurate content moderation decisions (see: https://transparency.fb.com/en-gb/policies/improving/content-actioned-metric/). Further, in a separate survey we conducted, real content moderators (who came from various social media platforms) tended to agree this decision process was an accurate reflection of the decisions they make in their own work. These data are available at: https://osf.io/j3td9/files/szyjt.

#### Intrusion monitoring task

We described intrusions to participants as any involuntary recollections (i.e., that come to mind with no conscious initiation or effort) they had of the images they viewed. We measured intrusion frequency by asking participants to press a key each time they experienced an image-related intrusion while they read unrelated, neutral science articles for five minutes (e.g., Takarangi et al., 2022). After the reading task, participants rated their intrusions on a 7-point scale (*1 = not at all*,* 7 = extremely).* Four items measured involuntariness (e.g., *The images I viewed earlier came to mind spontaneously*), retrieval intent (e.g., *I intended to bring the images I viewed earlier to mind*), and retrieval ease (e.g., *The images I viewed earlier came to mind effortlessly*). We used these items to determine whether the intrusions participants reported were indeed involuntary. The other six items measure problematic intrusion characteristics (i.e., intrusiveness, unwantedness, vividness, distress, emotional intensity, valence; Oulton et al., 2018). Participants then briefly described the content of their intrusions, allowing us to check whether they only reported intrusions of the study images. In line with our pre-registration, we disregarded the intrusion data for three participants whose responses indicated they misunderstood the intrusion monitoring task.

#### Positive and negative affect schedule (PANAS)

We assessed participants’ change in positive and negative affect from pre- to post-simulation using the 20-item PANAS (Watson et al., 1988). Participants indicated on a 5-point scale (*1 = very slightly or not at all*,* 5 = extremely*) how they felt on each of the 10 positive and 10 negative mood adjectives. Scores on the affect scales range from 10 to 50, and higher scores on either scale indicate higher negative or positive affect. The negative affect subscale (Cronbach’s α = 0.91) and the positive affect subscale (α = 0.91) demonstrated good internal consistency in the current study.

#### State-trait anxiety inventory-state scale short form (STAI-S-6)

We measured change in state anxiety from pre- to post-simulation using the 6-item STAI-S (Fioravanti-Bastos et al., 2011). Participants rated how they felt in the present moment for items such as “I feel comfortable” or “I feel tense” on a 4-point scale (*1 = not at all*,* 4 = very much*). Scores can range from 6 to 24, with higher scores indicating higher anxiety. This scale had excellent internal consistency in the current study (α = 0.94).

#### Effortful imagination

To assess whether the modified (blurred, greyscale) conditions would lead participants to try and imagine the details or colors of the images, we asked participants in these conditions how much effort they put into imagining the details of the blurred images or the color of the greyscale images (*1 = no effort at all*,* 4 = a lot of effort*). We also asked these participants how clear and vivid the details (blurred condition) or colors (greyscale condition) of the images were in their mind (*1 = not at all*,* 4 = extremely clear*,* as if they were unblurred/actually in color).*

#### Self-location

To assess whether the blurred or greyscale images might lead participants to feel more distant from the images compared to viewing unmodified images, we used adapted versions of the four “self-location” items from the Spatial Presence Experience Scale (Hartmann et al., 2015; e.g., “I felt like I was actually there in the environment of the images”). The items are rated from 1 (*I do not agree at all*) to 5 (*I fully agree*).

#### Compliance and technical questions

We asked participants how closely they paid attention to the images, whether they left the study at any point (and if so, at what point they left the task and for how long), whether they had any technical difficulties, and what they believed the aim of our study was. We used participants’ responses to these questions to guide our exclusion process^2^ (detailed in our pre-registration: https://osf.io/6ytkj/overview).

#### Exploratory questions

In the interest of other research our lab is conducting on content moderation, we asked participants in both studies some exploratory questions about meaning making. We do not report these data here. We also included the 20-item *Posttraumatic Stress Disorder Checklist* (PCL-5; Weathers et al., 2013) but due to an error in our survey, only participants who experienced intrusions saw the PCL-5 and meaning questions. As such, we do not report the exploratory analysis we had planned for the PCL-5 here, but it is available in the supplementary analyses.

#### Procedure

To enter the study, participants had to complete a reCAPTCHA and pass an English proficiency test, an Ishihara colored dot plate test (Ishihara, 1918), and a culture check (i.e., respond “eggplant” to an image of an eggplant) to ensure linguistic consistency in our US sample. Participants read the study information and consent form and we warned them they would be viewing potentially distressing content and could withdraw from the study at any point. If participants consented to participate (i.e., provided informed consent), they then provided demographic information (e.g., gender, age) before reading some information about content moderators. Participants then read the guidelines and answered the six practice questions. Participants who responded incorrectly to more than two of the questions were ineligible to continue the study (*n* = 10, not otherwise counted as exclusions in this study). Eligible participants completed pre-task measures of state anxiety (STAI-S-6) and affect (PANAS) before reading the introduction to the content moderator simulation. Then, they were randomly allocated to one of the three image conditions (unmodified *n* = 103; greyscale *n* = 104, blur *n* = 102) and completed the content moderator simulation task. Following the simulation, participants completed the STAI-S-6 and PANAS again before continuing onto the intrusion monitoring task. They rated their intrusions overall and answered the exploratory questions. Finally, we debriefed and compensated participants. On average, the entire procedure took around 35 min to complete.

### Results and discussion

#### Positive and negative affect

To assess whether modifying (i.e., blurring or greyscaling) negative images can mitigate negative changes in affect associated with content moderation, we first examined changes in negative affect and positive affect, separately, from pre-task to post-task by image modification type using one-way ANOVAs. We calculated change in negative and positive affect by subtracting pre-task scores from post-task scores for each subscale. Recall we predicted that participants who viewed blurred or greyscaled images would have a smaller decrease in positive affect and a smaller increase in anxiety and negative affect from pre- to post-task compared to those who viewed the unmodified images, with a larger difference for the greyscale versus control conditions compared to the blurred versus control conditions.

**Fig. 1:**
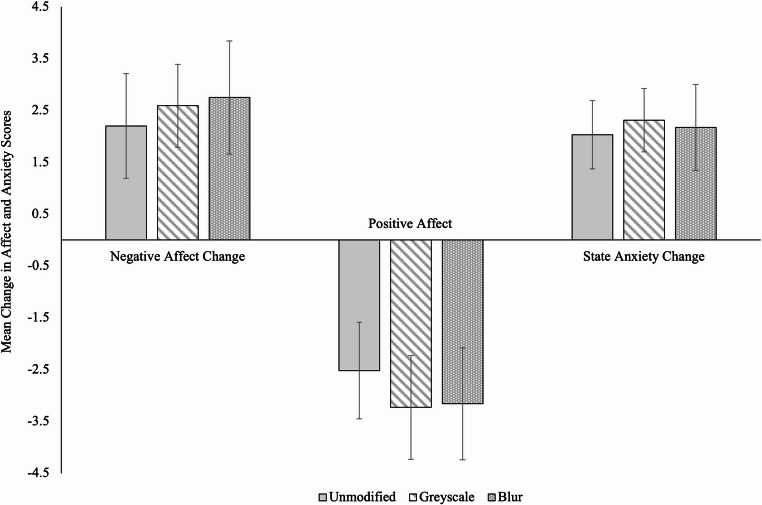
Mean change in negative affect, positive affect, and state anxiety by modification type, with 95% CIs

Turning first to negative affect, as shown in Fig. 1—where positive scores indicate an increase in negative affect over time—there was no effect of image modification type on the change in negative affect, *F*(2,306) = 0.31, *p* =.734, η_p_^2^ = 0.00, BF₁₀ = 0.05 (strong evidence for H₀).^3^ We conducted planned contrasts to test our specific hypotheses regarding differences between groups, but found no differences: that is, between the blur and greyscale conditions, greyscale and control conditions, and blur and control conditions, *t*s < 1 (see supplementary analyses for all planned contrasts). Our results therefore did not support our hypothesis. Given the idea that blurring and removing color should reduce arousal, we compared the control condition to the greyscale and blurred conditions, respectively, on two “high arousal” negative affect items from the PANAS (nervous, jittery; see Yik et al., 2011) separately. However, planned contrasts indicated no significant differences in nervous or jittery change scores between the control condition and either the blur or greyscale conditions (all *p*s > 0.60).

Turning next to positive affect, as shown in Fig. 1, our hypothesis was also not supported. That is, there was no statistically significant effect of image modification on the change in positive affect, *F*(2,306) = 0.59, *p* =.555, η_p_^2^ = 0.00, BF₁₀ = 0.06 (strong evidence for H₀), and planned contrasts revealed no differences between the groups, *ts* < 1.

#### State anxiety

To assess whether modifying (i.e., blurring or greyscaling) the visual features of negative imagery can mitigate anxiety associated with content moderation, we compared image modification type on change in state anxiety from pre-task to post-task with one–way ANOVAs. We calculated change in anxiety by subtracting pre-task scores from post-task scores. Recall we predicted that participants who viewed modified images would experience a smaller increase in state anxiety compared to participants who viewed unmodified images. As depicted in Fig. 1, image modification did not meaningfully prevent anxiety from increasing over time. Indeed, a one–way ANOVA comparing the effect of image modification type on change in state anxiety from pre- to post-task showed no statistically significant difference between the three conditions, *F*(2,306) = 0.16, *p* =.855, η_p_^2^ = 0.00, BF₁₀ = 0.04 (strong evidence for H₀). Further, planned contrasts revealed no differences between group comparisons, *ts* < 1. Therefore, our hypothesis was not supported. Together, these results indicate that regardless of image modification, the content moderator simulation increased participants’ negative affect and state anxiety and decreased positive affect (e.g., Takarangi et al., 2016; Takarangi et al., 2023).

#### Intrusion frequency and problematic characteristics

We investigated whether the two image modification strategies would influence content moderators’ susceptibility to intrusion frequency and problematic characteristics. We predicted that—if image modification works as intended—participants who viewed blurred or greyscaled graphic material would experience fewer intrusions and rate them lower on problematic intrusion characteristics than participants who viewed unmodified content. We first analyzed the intrusion retrieval intent and ease items to determine whether the intrusions were involuntary recollections and not deliberately or consciously brought to mind. This check was important because the intrusion-monitoring instructions (i.e., respond any time an involuntary memory of the images comes to mind) may have created an expectation to deliberately recall the images. Intent ratings (*M* = 1.78, *SD =* 1.26) were significantly lower than the scale anchor (‘7’) for intentional retrieval, *t*(228) = 21.41, *p* <.001, *d* = 1.42 (95% CI [1.23, 1.60]), and ease ratings (*M* = 5.38, *SD* = 1.43) were significantly higher than the scale anchor (‘1’) for low retrieval ease, *t*(228) = 56.95, *p* <.001, *d* = 3.76 (95% CI [3.39, 4.13]). These results suggest that participants typically did not deliberately or consciously bring their intrusions to mind.

Next, to ensure involuntariness did not differ by condition, we compared intrusion retrieval intent and ease between the groups using a Welch’s ANOVA (intent) and a one–way ANOVA (ease). There were no statistically significant differences between the groups for retrieval intent, Welch’s *F*(2,142.31) = 2.01, *p* =.137, η_p_^2^ = 0.02, BF₁₀ = 0.24 (moderate evidence for H₀), or retrieval ease, *F*(2,226) = 0.11, *p* =.896, η_p_^2^ = 0.00, BF₁₀ = 0.05 (strong evidence for H₀), indicating that the involuntary nature of the intrusions did not differ depending on whether the images were unmodified, blurred, or greyscaled.

**Fig. 2:**
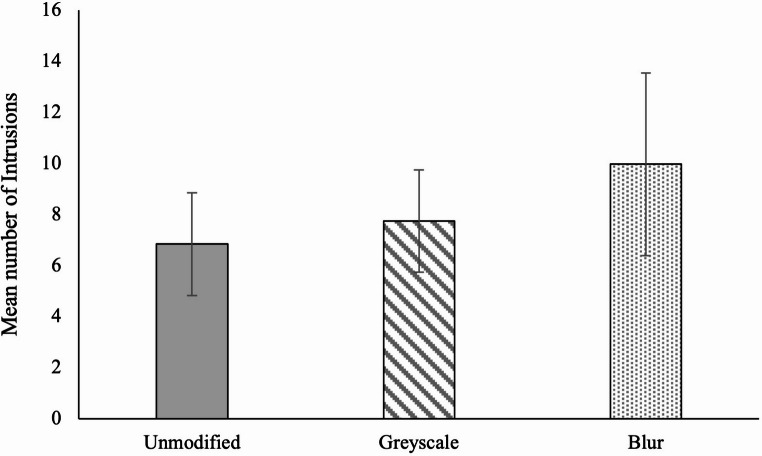
Mean number of intrusions reported by modification type, with 95% CIs

We next tested our main hypothesis. Figure 2 shows that the unmodified condition experienced fewer intrusions than both modified image conditions. However, when we ran a one–way ANOVA, we found no statistically significant differences in intrusion frequency between the conditions, *F*(2,306) = 1.16, *p* =.314, partial η_p_^2^ = 0.01, BF₁₀ = 0.10 (moderate evidence for H₀). That is, the modification strategies did not meaningfully change the number of intrusions participants experienced. Further, planned contrasts revealed no differences between group comparisons, *ts* < 1.5. These results therefore do not support our hypothesis.

Although it was not pre-registered, we also ran a negative binomial regression model, more suitable for count data (Coxe et al., 2009). Condition did not account for significant variance in the number of intrusions participants reported, likelihood ratio: χ2 (2) = 2.96, *p* =.228. Further, pairwise comparisons revealed that neither the greyscale condition, B = 0.12, SEB = 0.21, Exp (B) = 1.13 (95% CI [0.75, 1.69]), *p* =.550, nor the blur condition, B = 0.35, SEB = 0.21, Exp (B) = 1.42 (95% CI [0.94, 2.12]), *p* =.092, significantly differed from the control condition, or from each other, B = 0.22, SEB = 0.21, Exp (B) = 0.80 (95% CI [0.53, 1.20]), *p* =.275.

**Fig. 3:**
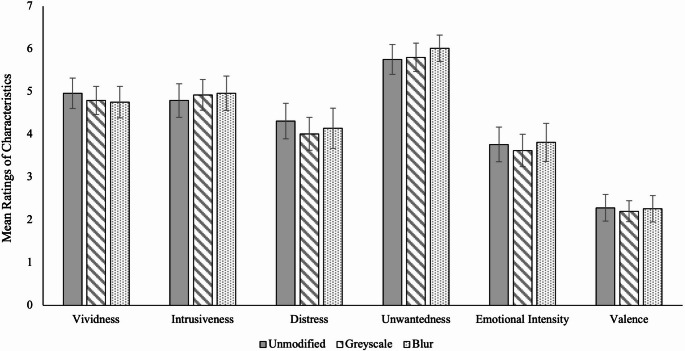
Mean levels of problematic intrusion characteristics by modification type, with 95% CIs

We next examined whether blurring or greyscaling the images would intensify problematic intrusion characteristics relative to unmodified images. To assess whether image modification type affected how problematic the intrusions were, we ran a one–way ANOVA on each of the six problematic intrusion characteristics items. Aligning with what we found for intrusion frequency, Fig. 3 shows there was no statistically significant difference between conditions for any of the problematic intrusion characteristics, all *F*s < 1, BF₁₀ range = 0.05–0.08 (strong evidence for H₀). In other words, neither blurring nor greyscaling exacerbated problematic intrusion characteristics. Planned contrasts also revealed no differences between group comparisons, *ts* < 1.08. Our hypothesis that participants who viewed modified images would rate their intrusions lower on problematic characteristics compared to participants who viewed unmodified images was therefore not supported.

#### Effortful imagination

Recall we included items to identify the extent to which image modification prompted participants to effortfully imagine the images, or affected how clear and vivid the images were in participants’ minds. Participants were not instructed to imagine the details or color of the images, so any effortful imagination arose naturally. Participants in the greyscale condition put “some” effort into imagining the colors of the images (*M* = 2.80, *SD =* 0.97), while participants in the blurred condition put “a lot” of effort into imagining the details of the images (*M* = 3.76, *SD =* 0.47). These ratings possibly indicate that blurring prompted a deeper level of effortful imagination than greyscaling, and potentially explaining why the blur group experienced more—albeit not significantly more—intrusions. However, differences in item wording across conditions (imagining *color* for greyscale versus *detail* for blurring) preclude strong conclusions about an underlying mechanism for our findings. Participants in the blurred condition reported that the imagined details of the images were “reasonably” clear and vivid in their mind (*M* = 2.93, *SD =* 0.67), and participants in the greyscale condition reported that the imagined colors of the images were between “somewhat” and “reasonably” clear and vivid in their mind (*M* = 2.57, *SD =* 0.93). That is, participants who viewed the blurred images reported descriptively more vividness and clarity in the imagined details of the images. Again, blurring may have prompted a deeper level of effortful imagination than greyscaling, but differences in item wording across conditions limit interpretation. Another caveat to these exploratory findings is that we did not ask the control group to report on how vividly they imagined the missing details or colors of the images—this question was not applicable to them. We address this limitation in Study 2 by including an equivalent imagination question for the control group to establish how vivid/clear the images were in participants’ minds when the details and color were actually present.

#### Self-location

The self-location questions aimed to determine whether image modification changed how immersed versus distant participants felt from the images. We expected that participants who viewed blurred or greyscale images would feel more distant from the images than participants who viewed unmodified images. However, we found no difference between the groups, *F*(2,306) = 1.01, *p* =.364, η_p_^2^ = 0.01, BF₁₀ = 0.13 (moderate evidence for H₀). That is, participants who viewed unmodified (*M* = 2.00, *SD =* 1.10), greyscale (*M* = 2.06, *SD =* 1.14), and blurred (*M* = 2.22, *SD =* 1.28) images, on average, did not agree with feeling physically present in the images. These results may support the effortful imagination results; perhaps participants who viewed modified images imagined the missing details in their mind sufficiently enough to feel equally immersed in the images as participants who viewed unmodified images. Alternatively, participants may not have fully engaged with the questions and responded arbitrarily across both groups.

Together, our results indicate that modifying graphic imagery by blurring or greyscaling in a content moderator simulation did not influence state anxiety, positive affect, negative affect, or intrusion frequency and problematic characteristics. Further, it appears that removing aspects of distressing content such as the color, or obfuscating details through blurring, may not be effective for reducing negative psychological outcomes because people experience the images in a similar way to how they would if they had seen them in color or unblurred. Our findings for positive affect diverge from previous research, where greyscaling and blurring increased content moderators’ positive affect compared to viewing unmodified images (Karunakaran & Ramakrishan, 2019). We suspect this discrepancy is because our participants were blinded to the idea that greyscaling or blurring might reduce psychological harm and had not viewed unmodified images first—unlike Karunakaran and Ramakrishan’s participants, who may have expected image modifications to improve their experience, thereby influencing affect through expectancy effects (Kirsch, 2019) or demand characteristics. Because real content moderators can choose to use image modifications—potentially mitigating negative outcomes through expectancy effects—we aimed to replicate this approach in Study 2.

## Study 2

Study 2 replicated Study 1 with several key modifications. First, participants chose the format in which they wanted to view the images. Second, participants in *all* conditions rated how clear and vivid the images were in their minds “*now”* (i.e., after the task). Following the simulation, we also asked participants *why* they chose the image format they did and how their chosen format influenced their experience of the task. Further, participants rated how helpful they thought their chosen format was for reducing the impact of the images and how helpful it was for categorizing the images accurately (to evaluate whether their perceptions were consistent with the effects we expected for each format).

Our primary interest in Study 2 was in whether the content moderator simulation would impact participants differently depending on whether they chose *any modification* or *no modification*. If choosing to use an image modification strategy is beneficial, we expected that participants who chose to view modified images (vs. those who chose to view unmodified images) would have a smaller decrease in positive affect and increase in anxiety and negative affect from pre- to post-task, experience fewer intrusions, and rate their intrusions lower on problematic characteristics. To explore potential differences in outcomes between participants who self-selected into the blur versus greyscale conditions, we conducted additional exploratory analyses between these groups.

### Method

#### Participants

Based on pilot testing (*n* = 46), we expected that at least 50% of people would choose to view unmodified images, and the remaining participants would choose greyscale or blur (at roughly equal rates). As such, we powered this study to analyze two groups: modified (greyscale/blur) versus unmodified. At 80% power, to detect *d* of at least 0.40, Brysbaert (2019) recommends a minimum of *n* = 100 participants per group, but *n* = 110 participants per group to obtain moderate evidence for the null hypothesis (BF < 0.33), for a two independent groups design. Therefore, we aimed to collect at least 110 participants for each group (unmodified; modified).

We collected data from 378 participants in 2024 to achieve a sample of 110 in the modified condition—70% of participants in the main study chose to view unmodified images, with only 14% choosing blur and 16% choosing greyscale. We recruited all participants from MTurk. In line with our pre-registration, we excluded data from 11 participants who provided an incorrect answer on any of the three neutral images that did not violate any ‘guidelines’. Similarly, we excluded data from two participants who provided an incorrect answer to more than three of the 14 images clearly containing violating content. Our final sample of 365 participants ranged from 22 to 81 years of age (*M* = 41.82) and were mostly women (~ 59%) or men (~ 39%), with the remainder of the sample indicating they were non-binary or that they preferred not to say. Seventeen participants indicated that they had experience as a content moderator (~ 5%), but again, participants may have interpreted content moderation more broadly than graphic imagery moderation.

#### Main task and measures

The Content Moderator Simulation (images, training, decisions), intrusion monitoring task, anxiety and affect measures, self-location questions, and compliance and technical questions^4^ were all the same as in Study 1.

## Choice questions

We asked participants why they chose the image format they did (open text box), and how their chosen format influenced their experience of the task (open text box). Participants also rated how helpful they thought their chosen format was for reducing the impact of the images and how helpful it was for categorizing the images accurately (both rated on a scale from *1 = not helpful at all*, to *4 = extremely helpful*).

### Effortful imagination

The effortful imagination questions were the same as in Study 1. We added an extra question: participants in *all* conditions rated how clear and vivid the images were in their minds *now* (after the task).

#### PTSD

To measure participants' PTSD symptoms in relation to their previous life experiences, participants were asked to bring their worst stressful or traumatic event to mind. They then identified whether (yes/no) during this event they were exposed to death, threatened death, actual or threatened serious injury, or actual or threatened sexual violence, in any of the following ways: (a) Direct exposure; (b) Witnessing the trauma; (c) Learning that a relative or close friend was exposed to a trauma; (d) Indirect exposure to aversive details of the trauma, usually in the course of professional duties (e.g., first responders, medics). All participants were then asked to respond to each of the 20 items on the PTSD checklist for DSM-5 (PCL-5; Weathers et al., 2013) in relation to their most stressful or traumatic event. Participants indicated on a 5-point scale (0 = not at all, to 4 = extremely) how much in the past month they have been for example “having difficulty concentrating” or “avoiding memories, thoughts, or feelings related to the stressful experience?”.

#### Exploratory questions

In the interest of other research our lab is conducting on content moderation, we asked participants about any mistakes they believed they made on the image decisions. We do not report these data here.

#### Procedure

Our procedure was the same as in Study 1, but following the introduction to the content moderator simulation, we told participants that content moderators can choose to view images in one of three ways: as they are (i.e., unmodified images, in color), with a blur, or in greyscale. Participants then chose their preferred format (unmodified *n* = 255; modified *n* = 110 [greyscale *n* = 59; blur *n* = 51]) before completing the content moderator simulation in that format. Following the intrusion task, participants answered the questions about their format choice. All participants then completed the PTSD questions.

### Results and discussion

Results for the PCL-5 and the self-location questions appear in the supplementary analyses.

#### Positive and negative affect

**Fig. 4:**
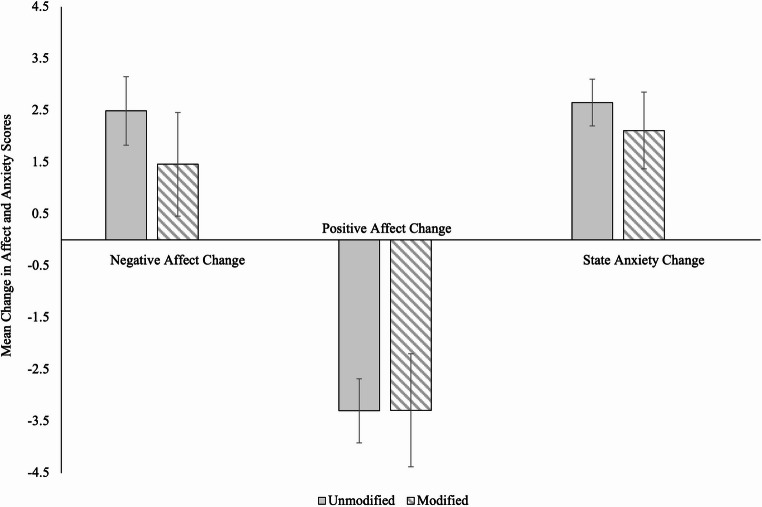
Mean change in negative affect, positive affect, and state anxiety by modification choice, with 95% CIs

To assess whether *choosing* to view modified (i.e., blurred or greyscaled) images can mitigate negative changes in affect associated with content moderation, we first examined changes in negative affect and positive affect, separately, from pre-task to post-task between participants who chose to view modified versus unmodified images using independent samples *t*-tests. Turning first to negative affect, as shown in Fig. 4, there was no effect of modification choice on change in negative affect, *t*(363) = −1.59, *p* =.113, *d* = −0.18 (95% CI [−0.40, 0.04]), BF₁₀ = 0.41 (anecdotal evidence for H₀). We also found no differences when comparing the change in negative affect between participants who chose to view greyscale (*M* = 1.24, *SD* = 7.52) versus blurred (*M* = 1.73, *SD* = 4.51) images, *t*(108) = 0.40, *p* =.686, *d* = 0.08 (95% CI [−0.30, 0.45]), BF₁₀ = 0.22 (moderate evidence for H₀).

Turning next to positive affect, displayed again in Fig. 4, choosing to view modified images did not prevent positive affect from decreasing relative to choosing to view unmodified images, *t*(363) = 0.01, *p* =.990, *d* = 0.00 (95% CI [−0.22, 0.22]). Similarly, participants who chose to view greyscale (*M* = −3.58, *SD* = 6.55) versus blurred (*M* = −2.96, *SD* = 4.70) images experienced a similar decrease in positive affect, *t*(108) = 0.56, *p* =.578, *d* = 0.11 (95% CI [−0.27, 0.48]), BF₁₀ = 0.23 (moderate evidence for H₀). Overall, our results are consistent with our findings in Study 1 and suggest that the moderation simulation has a similar emotional impact regardless of whether a person chooses to view the images in a modified or unmodified form.

#### State anxiety

We compared change in state anxiety from pre-task to post-task between participants who chose to view modified versus unmodified images. As depicted in Fig. 4, choosing to view modified images did not meaningfully prevent anxiety from increasing over time. That is, participants who chose to view unmodified images and participants who chose to view modified images experienced a similar increase in state anxiety, *t*(363) = −0.26, *p* =.794, *d* = −0.03 (95% CI [−0.25, 0.19]), BF₁₀ = 0.13 (moderate evidence for H₀). Similarly, the change in state anxiety did not differ between participants who chose to view greyscale images specifically (*M* = 4.63, *SD* = 3.81) and participants who chose to view blurred images (*M* = 4.33, *SD* = 3.47), *t*(108) = −0.42, *p* =.675, *d* = −0.08 (95% CI [−0.46, 0.29]), BF₁₀ = 0.22 (moderate evidence for H₀). Again, these results align with our findings from Study 1 and suggest that image modification is unsuccessful at mitigating anxiety, even when a person chooses to use an image modification strategy.

#### Intrusion frequency and problematic characteristics

We first analyzed the intrusion retrieval intent and ease items. Intent ratings (*M* = 1.99, *SD =* 1.42) were significantly lower than the scale anchor (‘7’) for intentional retrieval, *t*(286) = 23.82, *p* <.001, *d* = 1.41 (95% CI [1.24, 1.57]), and ease ratings (*M* = 5.29, *SD* = 1.31) were significantly higher than the scale anchor (‘1’) for low retrieval ease, *t*(286) = 68.68, *p* <.001, *d* = 4.05, (95% CI [3.70, 4.40]), indicating participants typically did not deliberately or consciously bring their intrusions to mind.

Next, we compared intrusion retrieval intent and ease between the groups. There was no statistically significant difference between the groups for retrieval ease, *t*(285) = −1.60, *p* =.112, *d* = −0.20 (95% CI [−0.45, 0.05]), BF₁₀ = 0.46 (anecdotal evidence for H₀), and a marginal difference between groups for retrieval intent, *t*(285) = 1.97, *p* =.050, *d* = 0.25 (95% CI [0.00, 0.50]), BF₁₀ = 0.86 (anecdotal evidence for H₀). Specifically, participants who viewed modified images (*M* = 2.23, *SD* = 1.50) were, on average, more intentional about bringing the images to mind than participants who viewed unmodified images (*M* = 1.88, *SD* = 1.37). Although the intrusions in this condition were *more* intentional, the average was still at the low end of the scale, indicating they were unintentional overall.

**Fig. 5:**
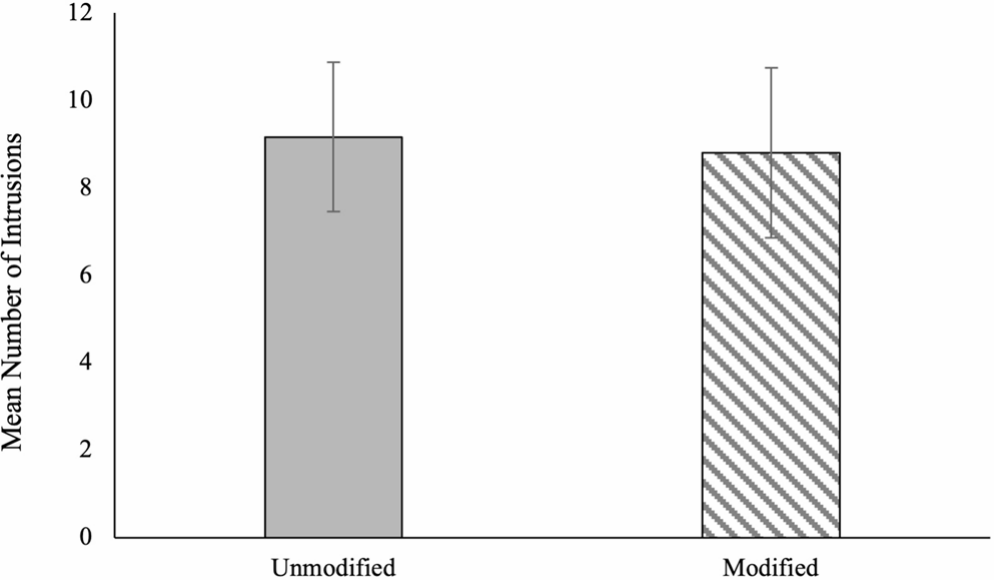
Mean number of intrusions reported by modification choice, with 95% CIs

We next tested whether choosing to view modified images would result in less frequent and problematic intrusions. As shown in Fig. 5, participants who chose to view unmodified (*M* = 9.16, *SD* = 13.88) images experienced a similar number of intrusions to participants who chose to view modified images (*M* = 8.80, *SD* = 10.28), *t*(363) = −0.24, *p* =.809, *d* = −0.03 (95% CI [−0.25, 0.20]), BF_10_ = 0.13 (moderate evidence for H_0_). Within the modified condition, participants who viewed greyscale images (*M* = 9.64, *SD* = 11.33) experienced a similar number of intrusions to participants who viewed blurred images (*M* = 7.82, *SD* = 8.94), *t*(108) = −0.93, *p* =.357, *d* = −0.18 (95% CI [−0.55, 0.20]), BF₁₀ = 0.30 (moderate evidence for H₀).

We also ran a negative binomial regression model. Condition did not account for significant variance in the number of intrusions participants reported, likelihood ratio: χ2 (1) = 0.06, *p* =.799. That is, the modified condition *p* did not significantly differ from the unmodified condition, B = 0.04, SEB = 0.16, Exp (B) = 1.04 (95% CI [0.76, 1.40]), *p* =.798. These findings align with our findings from Study 1 and suggest that viewing modified images does not influence how many image-related intrusions a person experiences, even when they choose to use an image modification strategy.

**Fig. 6:**
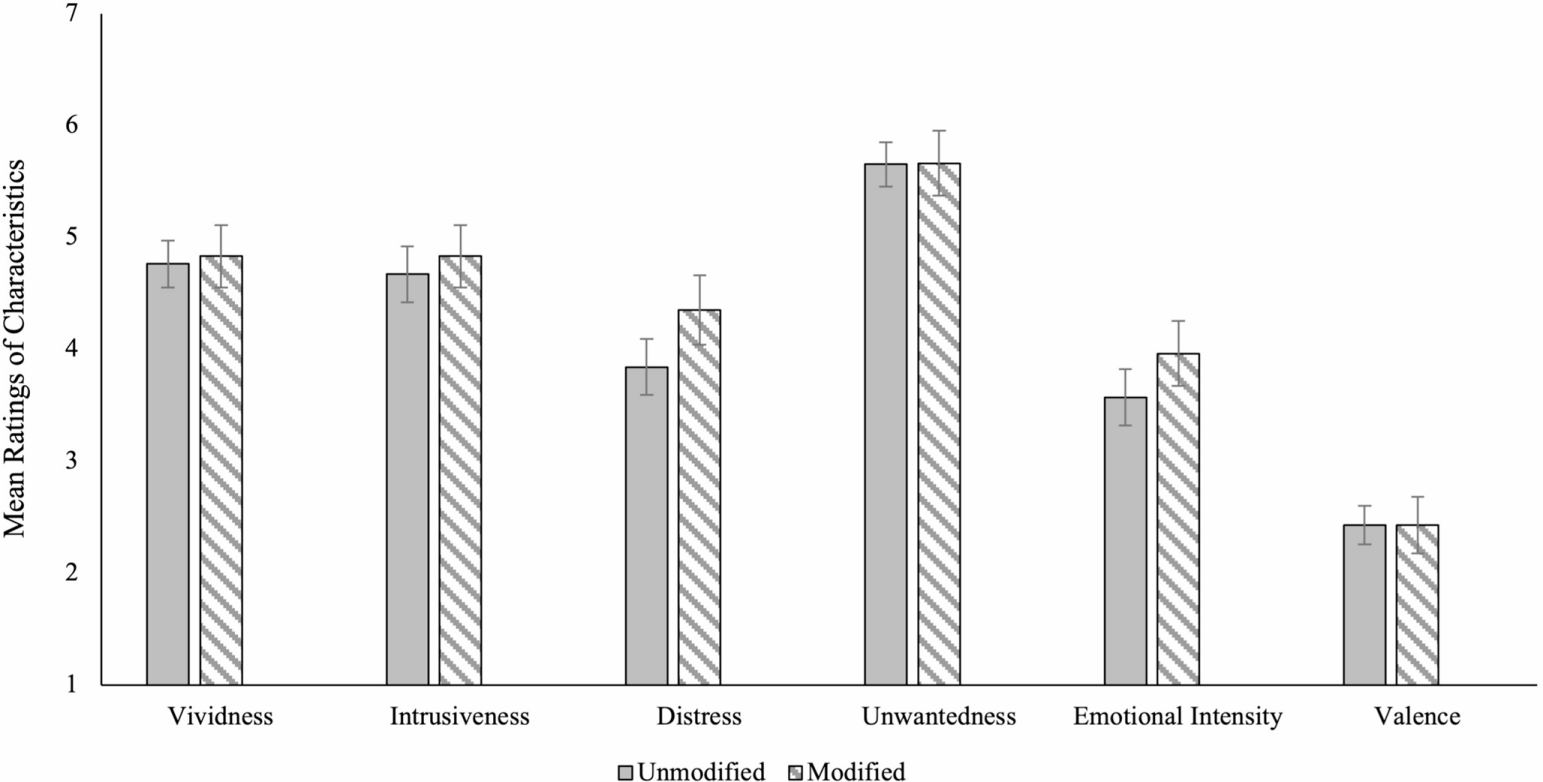
Mean levels of problematic intrusion characteristics by modification choice, with 95% CIs

Regarding intrusion characteristics, we examined whether choosing to view modified images would reduce the intensity of problematic intrusion characteristics relative to unmodified images. Figure 6 shows that there was no statistically significant difference between conditions for all problematic intrusion characteristics except for intrusion distress; participants who chose to view modified images rated their intrusions as more distressing than participants who chose to view unmodified images, Welch’s *t*(204.69) = 2.51, *p* =.013, *d* = 0.31. These results suggest that viewing modified images (by choice) does not mitigate the intrusiveness, vividness, unwantedness, emotional intensity, or negativity of intrusions, but may exacerbate the distress associated with those intrusions. Within the modified condition, there were no significant differences for intrusion characteristic ratings between participants who viewed greyscaled images and participants who viewed blurred images, *t* range = 0.43–1.48, BF₁₀ range = 0.24–0.57 (moderate to anecdotal evidence for H₀).

#### Effortful imagination

Paralleling Study 1, participants who chose to view greyscale images put “some” effort into imagining the colors of the images (*M* = 3.02, *SD =* 1.03), while participants who chose to view blurred images put “a lot” of effort into imagining the details of the images (*M* = 3.59, *SD =* 0.61). Similarly, participants who chose greyscale images reported that the imagined colors of the images were “somewhat” to “reasonably” clear and vivid in their mind (*M* = 2.46, *SD =* 0.99), and participants who chose blurred images reported that the imagined details of the images were “reasonably” clear and vivid in their mind (*M* = 2.92, *SD =* 0.74).

Recall that this time we also asked *all* participants how clear and vivid the images were in participants' minds *after* the task. Participants who viewed unmodified images (*M* = 3.23, *SD* = 0.70) reported that the images were significantly more clear and vivid in their mind than participants who viewed modified images (*M* = 2.84, *SD* = 0.77), *t*(363) = −4.77, *p* <.001, *d* = −0.54 (95% CI [−0.77, −0.32]).

These results could suggest that image modification—especially blurring—prompts a level of imagination that is sufficient to create a “reasonably” clear and vivid picture in people’s minds. Although this picture is not quite as clear as if the image was unmodified, our results overall suggest that the images impact people similarly, regardless of modification. At the same time, the differences in our item wording across conditions mean that the present data cannot establish effortful imagination as a specific underlying mechanism for the similar outcomes across modification conditions.

#### Reason for choice

We asked participants to explain why they chose the image format they did and how they believed their chosen format influenced their experience. The lead author (SL) coded responses according to clear themes that emerged in the responses. In some cases, a participant's response contained two themes, and in others the response was not codable (e.g., “Was simple”).^5^ Coding these data revealed that—among participants who chose unmodified images—half (51%) mentioned they wanted their decisions about the images to be accurate. Participants also cited wanting to see the images in their true form (12.6%), having tolerance for graphic imagery (12.2%), preferring clarity or being curious about the image (10.2%), and wanting to experience the full impact of the images (5.1%). Some participants also reported that they did not want the images to be subject to their imagination (2%), or that they were against censorship (1.2%). Participants who chose greyscale mostly reported that they believed the greyscale images would be less impactful (52.5%) and/or less graphic (30.5%). Participants also explained that they believed the greyscale images would be clearer and easier to judge than the blurred images (52.5%). For participants who chose to view blurred images, they also explained that they believed the blurred images would be less impactful (51%) or less graphic (45.1%).

#### Perceived helpfulness of strategies

Recall that we asked participants how helpful they believed their chosen format was for reducing the impact of the images and for categorizing the images accurately. Unsurprisingly, participants who chose unmodified images (*M* = 3.62, *SD* = 0.68) rated their choice as more helpful for *accurately categorizing the images* than participants who chose modified images (*M* = 2.60, *SD* = 0.91), Welch’s *t*(162.92) = −10.60, *p* <.001, *d* = −1.28. On the flip side, participants who chose modified images (*M* = 2.95, *SD* = 0.81) rated their choice as more helpful for *reducing the impact of the images* than participants who chose unmodified images (*M* = 1.73, *SD* = 1.14), Welch’s *t*(287.60) = 11.66, *p* <.001, *d* = 1.24. These results quantitatively reflect participants’ motivations behind their choice to use image modification or not and the balance between participants’ desire for accuracy and their desire to mitigate emotional impact. That is, those who chose unmodified images emphasized accuracy and viewing images in their true form, while those who opted for modified images prioritized reducing the graphic nature or emotional intensity of the images.

#### Influence of choice on task

We asked participants to explain how they thought their chosen format influenced their experience of the task. Participants who chose to view unmodified images most commonly reported that this choice made their performance more accurate (34.5%). However, participants also reported that this choice made the task more distressing (21.2%), intense (10.6%), or real (8.2%), or made the images more memorable (7.7%). Some participants reported that choosing to view unmodified images had no impact on the task (12.2%). Participants who chose to view greyscale images reported most commonly that the greyscale did make the images less impactful (67.8%). However, some participants noted that they believed they were less accurate in their decisions (11.9%), or that there was no impact of the strategy on the task at all (10.2%). Some participants reported that greyscale was generally helpful (6.8%), made them more accurate (1.7%), or made the task more distressing (1.7%). The participants who chose to view blurred images also mainly reported that this strategy made the task less impactful (51%). However, participants also noted that the blur made them less accurate (15.7%) and prompted them to imagine the details of the images (11.8%). Some participants reported that the blur was generally helpful (5.9%), had no impact (3.8%), made the images more memorable (2%), or their responses more accurate (2%).^6^

## General discussion

In these studies, we investigated the effectiveness of a strategy Facebook currently employs for content moderators: moderators can choose to blur or greyscale content. Given that blurring out distressing details and removing colors may reduce the negative impact of viewing graphic images, we sought to determine whether, compared to viewing unmodified images, greyscaling and blurring could prevent positive affect from declining, prevent negative affect and anxiety from increasing, and reduce intrusion frequency and their associated problematic characteristics. We took two approaches to answering this question: one that maximized experimental control (Study 1: random allocation to image modification condition) and one that maximized ecological validity (Study 2: self-selection into image modification condition).

Across both studies, we found that neither blurring nor greyscaling meaningfully reduced *any* negative effects of viewing negative images. That is, neither removing color or blurring the images prevented anxiety or negative affect from increasing nor positive affect from declining, even when participants *chose* to view modified images. These results contradict the previous research on image modification. Recall that Besançon et al. (2020) found stylizations reduced the repulsiveness of surgical images, and Karunakaran and Ramakrishan (2019) found that greyscaling *increased* moderators’ positive affect while blurring *decreased* their positive affect. We expect the discrepancy between our findings relates to these studies using within-subjects designs, where participants’ emotional experiences in the modified conditions were likely influenced by their experience in the unmodified condition (e.g., contrast effects). Further, the within-subjects studies had limited experimental control and potential demand effects.

However, the discrepancy between our findings and previous research may also reflect limitations in how we implemented the visual modifications. Because we applied these modifications uniformly (i.e., fixed blur and greyscale), rather than allowing for different levels or application of blurring and greyscaling (e.g., increasing blur via a slider or hovering over a specific part of the image to blur it; Das et al., 2020) or targeting particular visual features (e.g., texture, hue, lines and edges; Besançon et al., 2020), it is possible our manipulation did not target the mechanisms that have reduced arousal in other approaches. Moreover, we did not include an independent manipulation check to assess whether our manipulations reduced the salience of potentially important cues (e.g., blood, wounds, facial expressions)—a limitation that future work should address while preserving the image details necessary for accurate decision-making. Importantly however, our standardized image modifications provided high experimental control. As such, although we cannot conclude that all modification strategies are ineffective, these particular implementations, under these conditions, do not attenuate negative psychological outcomes.

Our results also contradicted the idea that expectancy effects might lead participants who selected modified images to fare better in the content moderator simulation. Perhaps making a single decision regarding image modification (i.e., choosing to blur or greyscale all images) was not enough to make participants feel they had control over how they viewed the images. Recall that participants in Das et al.’s (2020) study had control over the blur on each image—likely accounting for their increased positive affect compared to those with no control. Notably, although participants who chose to view modified images did not differ from participants who chose to view unmodified images (except for rating their intrusions as *more* distressing and intentional), they did *believe* that the task impacted them less, according to the qualitative data we collected. Taken together, participants who chose to view modified images likely expected to fare better, resulting in a belief that they did fare better, despite their experience being similarly negative to those who viewed unmodified images. These findings highlight the limitations of assessing strategy effectiveness based on anecdotal or subjective reports and underscore the importance of using more objective data to assess impact accurately.

The divergence between participants’ subjective beliefs and their psychological outcomes underscores a broader policy concern in content moderation; namely, the trade-off between wellbeing and performance. In Study 2, many participants reported choosing unmodified images because they believed this would support accurate decision-making. Although performance outcomes were not a preregistered focus of the present studies, we conducted exploratory checks using a consensus-based threshold to estimate “accuracy” across modification conditions. We identified 14 images that at least 90% of participants across previous samples (*ns* 447–1,320) using variations of the same image set agreed violated guidelines. For each participant, we computed a consensus-based “accuracy” score reflecting the number of these 14 images for which they responded “yes” (i.e., violation present). Using this approach, consensus-based accuracy rates were similar across conditions in Study 1 (Control *M* = 13.56, Blur *M* = 13.45, Greyscale *M* = 13.43) and in Study 2 (Control *M* = 13.65, Blur *M* = 13.63, Greyscale *M* = 13.56). Although these analyses are exploratory and only a proxy for accuracy, this pattern suggests that image modifications did not influence participants’ violation decisions. Future research should directly examine the trade-off between harm mitigation and performance using preregistered accuracy metrics.

Regarding the difference we found between conditions for intrusion *distress*, it is likely that the subset of people who chose to view modified images did so because they were less willing to view graphic imagery—indeed, the qualitative data suggest participants chose modified images to reduce the contents’ impact and/or make it less graphic. As such, it may be that these people rated intrusive memories of the images as more distressing because they are more sensitive to disturbing content. Regarding intrusion intentionality, if participants who chose to view modified images are a more sensitive or cautious population, they may have engaged with the content in a more controlled or deliberate way, potentially increasing how intentional their intrusions felt.

A key limitation of our design is that we cannot determine whether the modified images themselves influenced participants’ experience, or whether the choice to view modified images influenced participants’ expectations and therefore their experience. Future research could measure participants’ expectations (e.g., for how their chosen format will influence their affect, anxiety, and intrusions), but disentangling choice and outcome through experimental manipulation is impossible for research investigating the effects of allowing people to *choose*. However, given that image modification had no effect in Study 1, it is likely that participants being able to choose modification accounts for the difference in intrusion distress we observed in Study 2. It is even possible that when participants’ expectations for the images being improved through modifications were not met, they experienced a contrast effect whereby the images seemed worse than they otherwise would have (Bridgland et al., 2019).

We suspect that there were no other differences between groups across our studies because participants may have experienced the modified images in their minds similarly to how they would have if they were unblurred or colored. Participants in the modified conditions reported imagining missing details or colors, particularly in the blur condition. This finding aligns with our hypothesis that blurring out details or removing color could encourage participants to imagine details beyond what was present in the image. However, our methodological approach limits causal conclusions regarding imagination and warrants future research to directly assess this as a mechanism in image modification outcomes.

## Constraints on generality

We designed these studies to inform harm-reduction strategies for professional content moderators, but our sample was drawn from U.S.-based MTurk workers, only 6% of whom had prior moderation experience. Using this sample limits the extent to which we can generalize our findings to professional moderators whose demographic profiles, training, workloads, and exposure histories may differ substantially. Although MTurk samples are typically WEIRD (Western, Educated, Industrialized, Rich, Democratic) and may not represent the full diversity of content moderators worldwide, they offer a practical and reasonably diverse online workforce. MTurk workers with no moderation experience could resemble new or prospective moderators, but our findings may not extend to experienced moderators, who may respond differently to image modification (e.g., Cook et al., 2022). We acknowledge these demographic and experiential limitations and recommend future studies recruit more diverse and professional samples where possible.

We aimed to preserve ecological validity with our content moderator simulation, and feedback from real content moderators (*n* = 14) who have reviewed our simulation suggest that it closely mirrors their work, supporting its validity as a reasonable analogue (Takarangi et al., 2026). However, we could not replicate several aspects of the content moderator experience. First, content moderators typically work under strict time constraints, spending less than 30 seconds on each piece of content (Newton, 2019b), and are expected to identify and remove violations at a 98% accuracy rate (Newton, 2019a). Second, real-world content moderation workplaces may be characterized by organizational supports, peer interaction, and formal wellbeing resources (however, we know engagement with, access to, and usefulness of resources and support is highly varied; Steiger et al., 2021; Takarangi et al., 2026). Third, our image stimuli did not capture the full range of real-world content, which includes videos and illegal content (e.g., child exploitation). Given the potential for time pressure, workload pacing, and video/audio stimuli to contribute to arousal and intrusion formation (e.g., Rendon-Velez et al., 2016; Rombold et al., 2016; Kulczycka et al., 2025), these missing aspects in our study further limit the generalizability of our findings from MTurk workers in a control setting to real-world content moderation. Further, our conclusions are limited to an *immediate* time frame; future research could test whether differences between modification conditions emerge over longer delays.

Although our simulation represents a comparatively less intense experience than what professional moderators experience, it also allowed us to test the core premise of this intervention: that removing surface-level visual detail reduces the impact of viewing negative content. If this foundational mechanism does not produce benefits—even under controlled conditions—we should question the effectiveness of this strategy with more graphic content, greater time pressure, and higher emotional and decisional stakes. Nonetheless, although controlled testing provides useful preliminary evidence about the potential limits of image modification, additional research with professional moderators is warranted to evaluate its effectiveness under real-world conditions.

## Conclusion

In conclusion, the mixed findings from previous research, alongside the results of the present study, suggest that further research is needed before social media platforms rely on image modification as a harm-reduction strategy. While modifying images by greyscaling or blurring may, on face-value, appear to reduce emotional distress, we found that participants experienced similarly negative effects, regardless of whether they chose to modify the images, or which modification strategy they used. Importantly, providing people with a single, overarching decision about image modification may not offer the same sense of control as allowing them to adjust the level of modification on each individual image, as seen in previous research. Future research should expand on this work using a professional content moderator sample and examine whether being able to choose modification on an image-by-image basis (a) reduces anxiety and intrusions, and (b) is a feasible strategy given the pressures within content moderation (e.g., time, accuracy). Overall, further research is needed to determine which intervention strategies genuinely protect content moderators.

## Supplementary Information

Below is the link to the electronic supplementary material.

Supplementary Material 1

Supplementary Material 2

## Data Availability

The materials we used and the data we collected from participants in our online studies are available on the Open Science Framework. Links to access the data and materials in addition to our pre-registrations are provided throughout our manuscript and are available via: https://osf.io/ck6ab/.

## Footnotes

1 For the null hypothesis, anecdotal = 1–0.3, substantial = 0.3–0.1, strong = 0.1–0.03, very strong = 0.03–0.01, decisive < 0.01.

2 For participants (*n* = 8) who we did not exclude but who indicated they left the study or did not pay close attention to the images (less than 4/7 on the rating scale), we reanalyzed our data without them, and the pattern of results for each of our analyses remained the same.

3 See supplementary analyses for descriptive statistics and one-sample *t*-tests (not pre-registered) testing change in positive affect, negative affect, and state anxiety from pre- to post-task for both studies. These analyses were always significant in the expected directions (e.g., the task led to higher negative affect).

4 We reanalyzed our data without participants (*n* = 10) who indicated they left the study, and the pattern of results for each of our analyses remained the same.

5 The percentage of non-codable responses was 5.9% for participants who chose unmodified images, 13.6% for those who chose greyscale images, and 3.9% for those who chose blurred images.

6 The percentage of non-codable responses was 7.1% for participants who chose unmodified images, 6.8% for those who chose greyscale images, and 15.7% for those who chose blurred images.
