# Supplementary material for: Evaluating image modification as a harm reduction approach in content moderation
Source: Psychol Res. 2026 Mar 26;90(2):62. doi: 10.1007/s00426-026-02241-5 (PMC13021741; doi:10.1007/s00426-026-02241-5)
Supplement: Supplementary file 1 — Supplementary Material 1 [file 426_2026_2241_MOESM1_ESM.docx]

**Online Resource 2 – Materials and Instructions**

This document includes the instructional materials used in both studies.

**Contents**

Information about Content Moderators ……………………………………………………….2

Content Guidelines ……………………………………………………………………………3

Guideline Comprehension Questions …………………………………………………………5

Introduction to Content Moderator Simulation ……………………………………………….7

Intrusion Task Instructions ……………………………………………………………………8

**Information about Content Moderators**

Content moderators are hired by platforms such as Facebook, Instagram, YouTube, Twitter, and others.

Content moderators perform an imperative task for these social media companies – they keep users safe by removing or putting sensitive or "warning" screens over content that violates a platform’s guidelines. Often, this content is distressing in nature (e.g., violence, child exploitation, animal abuse).

For the purpose of protecting users from this distressing content, as well as potentially viewing evidence for criminal activity, content moderators are required to have exceptional attention to detail and effective decision-making skills.

**Content Guidelines**

Next, we would like you to read some guidelines based on Facebook’s “community standards” for safe content. Some content is not allowed to be posted on Facebook and should be flagged for removal by the content moderator. Other content can be posted but only with a sensitive screen. 

Please take your time and read the guidelines carefully. You will be asked some questions about these guidelines that you will need to answer correctly to proceed with the study.

**TO BE REMOVED**
**Self Harm**

- Content that promotes, encourages, coordinates, or provides instructions for:
  - Suicide
  - Self-injury
- Content that depicts graphic self injury

**Hate Speech**

- Mocking the concept, events, or victims of hate crimes, even if no real person is depicted in an image
- Expressions of contempt (in written or visual form), which is defined as:
  - Self-admission to intolerance on the basis of protected characteristics, including, but not limited to racist and homophobic
- Segregation in the form of calls for action, statements of intent, aspirational or conditional statements, or statements advocating or supporting segregation

**Dead Bodies**

- Images of people or dead bodies in non-medical settings if they depict:
  - Dismemberment
  - Visible internal organs
  - Partial decomposition
  - Charred or burning people unless in the context of cremation
  - Victims of cannibalism

**Harm Against Animals**

- The following content involving animals:
  - Images depicting humans killing animals if there is no explicit manufacturing, hunting, food consumption, processing, or preparation context
  - Imagery of humans committing acts of torture or abuse against live animals
  - Imagery of animals showing wounds or cuts that render visible innards or dismemberment, if there is no explicit hunting, taxidermy, medical treatment, rescue or food consumption, preparation or processing context, or the animal is already skinned or with its outer layer fully removed

**Harm Against Property**

- Statements of intent, calls to action, representing, supporting, or advocating for harm against property that depicts, admits to, or promotes the following acts committed by the person posting or their associates:
  - Vandalism
  - Theft
- Positive statements about theft when committed by a third party

**PUT A WARNING SCREEN ON**
**Violent Death**

- Imagery that shows the violent death of a person or people by accident or murder.
- More specifically, imagery of the death of a person or people when there is not dismemberment, visible internal organs, decomposition, or charring, must have a screen added.

**Torture**

- Acts of torture committed against a person or people. For example:
  - Strangling
  - Beating
  - Restraining

**Guideline Comprehension Questions**

**Content Questions**

**1)** An image of a dead body in a non-medical setting must be removed from Facebook if it depicts:

**a.** Dismemberment

**b.** Visible internal organs

**c.** Partial decomposition

**d.** All of the above

**2)** Imagery that depicts the strangling, beating, or restraining of a person is considered .......... and must have a warning screen added to it.

**a.** Torture

**b.** Violent death

**c.** Hate speech

**d.** Self-injury

**3)** Content that depicts graphic self-injury must be removed from Facebook. What action is required for content that promotes, encourages, coordinates, or provides instructions for suicide or self-injury?

**a.** No action is required

**b.** It must, too, be removed

**c.** It must have a warning screen added

**d.** It must be flagged for criminal activity

**4)** Imagery depicting harm against animals must be removed from Facebook. Which of the following would need to be removed?

**a.** A human committing an act of torture or abuse against a live animal

**b.** A human killing an animal for no explicit reason or purpose

**c.** Imagery showing animals with wounds or cuts that render visible innards or dismemberment for no explicit reason or purpose

**d.** All of the above

**5)** Imagery of .......... posted by the perpetrator, or their associates, must be removed from Facebook.

**a.** Stalking

**b.** Vandalism

**c.** Harassment

**d.** Manipulation

**6)** Which of the following are a demonstration of hate speech and must be removed from Facebook?

**a.** Mocking victims of a hate crime

**b.** Expressions of racism

**c.** Statements supporting segregation

**d.** All of the above

**Introduction to Content Moderator Simulation**

It is important that content moderators have a thorough understanding of the Facebook policies so that they can correctly determine which content must be removed from Facebook and which content requires a warning screen.

You will now be shown some images and asked to decide whether the content of each image violates any of the policies you learned about earlier.

Note that some answers may be ambiguous, as is the case in a real content moderation role. Please try to answer the questions as best you can, keeping in mind the guidelines you just read.

**Intrusion Task Instructions**

Next, we would like you to read through two science articles.

Whilst reading, please indicate, via pressing the "X" key on your keyboard, the occurrence of any intrusions you have of the images you viewed in the content moderation task.

Intrusions are involuntary memories that come to mind without any conscious attempt to bring the memory to mind. These memories always pop to mind spontaneously, without any intentional attempt to remember them. You may have involuntary memories or recollections of the images you viewed earlier during this next reading task. If whilst reading one of the articles, an image from the previous task pops into your mind spontaneously, indicate this has occurred by pressing X.

Please also remember to press the X key EVERY TIME you experience an involuntary memory of the images you viewed earlier – even if you have already experienced the same memory. Do not press the X key for any intrusions you may have that ARE NOT of the IMAGES you viewed in the content moderation task.
