# Supplementary material for: Evaluating image modification as a harm reduction approach in content moderation
Source: Psychol Res. 2026 Mar 26;90(2):62. doi: 10.1007/s00426-026-02241-5 (PMC13021741; doi:10.1007/s00426-026-02241-5)
Supplement: Supplementary file 2 — Supplementary Material 2 [file 426_2026_2241_MOESM2_ESM.docx]

**Online Resource 1 - Supplementary Analyses**

This document includes additional statistical analyses for both studies.

**Contents**

Study 1: PCL-5 Results ……………………………………………………………………….2

Study 1 and 2: Descriptive Statistics for Affect and Anxiety ………………………………...3

Study 1 and 2: One Sample *t*-tests Results for Affect and Anxiety …………………………..5

Study 1: Planned Contrast Results ……………………………………………………………6

Study 2: PCL-5 Results …………………………………………………………………….....9

Study 2: Self-Location Question Results ……………………………………………………10

**Study 1: PCL-5 Results**

**Supplementary Table 1**

*Pearson Correlations Between PCL-5 Scores and Main Dependent Variables*

| **Variable** | ***r*** |  | ***p*** |
| --- | --- | --- | --- |
| Positive Affect | -.15 | * | .021 |
| Negative Affect | .21 | ** | .002 |
| State Anxiety | .19 | ** | .004 |
| Intrusion Frequency | .19 | ** | .005 |
| Intrusion intrusiveness | .28 | *** | <.001 |
| Intrusion distress | .33 | *** | <.001 |
| Intrusion vividness | .31 | *** | <.001 |
| Intrusion unwantedness | .17 | * | .012 |
| Intrusion emotional intensity | .35 | *** | <.001 |
| Intrusion valence | .03 |  | .602 |

*Note*. * *p* < .05, ** *p* < .01, *** *p* < .001

**Comparison Between Conditions**

Although not pre-registered, we checked whether participants scored similarly on the PCL-5 across conditions. There was no difference in PCL-5 scores between participants who viewed unmodified images (*M* = 18.67, *SD* = 16.65) greyscaled images (*M* = 17.94, *SD* = 14.22), or blurred images (*M* = 18.87, *SD* = 18.36), *F*(2, 229) = 0.07, *p* = .931.

**Descriptive Statistics for Positive and Negative Affect and State Anxiety**

**Supplementary Table 2**

*Means, Standard Deviations, Change Scores, and Effect Sizes for Positive and Negative Affect and State Anxiety at Pre-Task and Post-Task Within Each Condition in Study 1*

|  | **Time** | |  | **Cohen’s *d’s*** |
| --- | --- | --- | --- | --- |
| **Condition** | **Pre-task** | **Post-task** | **Change** | ***with 95% CI’s*** |
| **Positive Affect** |  |  |  |  |
| Unmodified | 28.61 (8.45) | 26.09 (8.69) | -2.52 (4.73) | -0.29 (-0.49, -0.10) |
| Greyscale | 28.79 (9.24) | 25.56 (9.14) | -3.23 (5.14) | -0.35 (-0.55, -0.15) |
| Blur | 28.09 (8.79) | 24.93 (8.80) | -3.16 (5.51) | -0.36 (-0.56, -0.16) |
| ***Total*** | 28.50 (8.81) | 25.53 (8.86) |  |  |
| **Negative Affect** |  |  |  |  |
| Unmodified | 14.06 (5.41) | 16.26 (6.60) | 2.20 (5.05) | 0.36 (0.16, 0.56) |
| Greyscale | 14.25 (5.37) | 16.84 (7.55) | 2.59 (4.64) | 0.40 (0.20, 0.60) |
| Blur | 14.75 (6.83) | 17.50 (8.23) | 2.75 (5.48) | 0.36 (0.16, 0.56) |
| ***Total*** | 14.35 (5.89) | 16.86 (7.48) |  |  |
| **State Anxiety** |  |  |  |  |
| Unmodified | 10.83 (4.39) | 12.85 (4.80) | 2.03 (3.33) | 0.44 (0.24, 0.64) |
| Greyscale | 11.11 (4.68) | 13.41 (5.53) | 2.31 (3.20) | 0.45 (0.25, 0.65) |
| Blur | 11.29 (5.27) | 13.46 (5.52) | 2.17 (4.16) | 0.40 (0.20, 0.60) |
| ***Total*** | 11.07 (4.78) | 13.24 (5.28) |  |  |

*Note.* Scores can range from 6 to 24 for State Anxiety, and 10 to 50 for Negative Affect.

**Supplementary Table 3**

*Means, Standard Deviations, Change Scores, and Effect Sizes for Positive and Negative Affect and State Anxiety at Pre-Task and Post-Task Within Each Condition in Study 2*

|  | Time | |  | Cohen’s *d’s* |
| --- | --- | --- | --- | --- |
| Condition | Pre-task | Post-task | Change | *with 95% CI’s* |
| **Positive Affect** |  |  |  |  |
| Unmodified | 29.27 (8.56) | 25.97 (8.55) | -3.30 (4.99) | -0.39 (-0.52, -0.26) |
| Modified | 26.12 (7.59) | 22.83 (7.78) | -3.29 (5.75) | -0.43 (-0.63, -0.24) |
| Greyscale | 26.42 (7.78) | 22.85 (8.02) | -3.58 (6.55) | -0.45 (-0.73, -0.17) |
| Blur | 25.76 (7.43) | 22.80 (7.58) | -2.96 (4.70) | -0.39 (-0.68, -0.11) |
| ***Total*** | 28.32 (8.39) | 25.02 (8.44) |  |  |
| **Negative Affect** |  |  |  |  |
| Unmodified | 13.98 (6.15) | 16.47 (7.75) | 2.49 (5.38) | 0.36 (0.23, 0.49) |
| Modified | 16.62 (7.74) | 18.08 (7.01) | 1.46 (6.28) | 0.20 (0.01, 0.39) |
| Greyscale | 17.56 (8.53) | 18.80 (7.12) | 1.24 (7.52) | 0.16 (-0.10, 0.42) |
| Blur | 15.53 (6.63) | 17.25 (6.85) | 1.73 (4.51) | 0.26 (-0.03, 0.55) |
| ***Total*** | 14.78 (6.77) | 16.96 (7.56) |  |  |
| **State Anxiety** |  |  |  |  |
| Unmodified | 10.48 (4.40) | 13.14 (4.88) | 2.65 (3.60) | 0.57 (0.44, 0.70) |
| Modified | 12.71 (4.59) | 14.82 (4.43) | 2.11 (3.93) | 0.47 (0.27, 0.67) |
| Greyscale | 12.95 (4.74) | 15.17 (4.53) | 2.22 (4.10) | 0.48 (0.20, 0.76) |
| Blur | 12.43 (5.45) | 14.41 (4.32) | 1.98 (3.75) | 0.40 (0.10, 0.70) |
| ***Total*** | 11.15 (4.57) | 13.64 (4.81) |  |  |

*Note.* Scores can range from 6 to 24 for State Anxiety, and 10 to 50 for Negative Affect.

**One Sample *t*-tests Results**

**Supplementary Table 4**

*One Sample t-test Results for Change Scores for Positive and Negative Affect and State Anxiety Across Both Studies*

|  | **Means and SDs** | **One Sample *t*-test** |
| --- | --- | --- |
| **Study 1** |  |  |
| Positive Affect | *M* = -2.97, *SD* = 5.13 | *t*(309) = -10.19, *p* < .001, *d* = -0.58 |
| Negative Affect | *M* = 2.51, *SD* = 5.05 | *t*(309) = 8.74, *p* < .001, *d* = 0.50 |
| State Anxiety | *M* = 2.17, *SD* = 3.58 | *t*(309) = 10.66, *p* < .001, *d* = 0.61 |
| **Study 2** |  |  |
| Positive Affect | *M* = -3.30, *SD* = 5.22 | *t*(364) = -12.06, *p* < .001, *d* = -0.63 |
| Negative Affect | *M* = 2.18, *SD* = 5.68 | *t*(364) = 7.34, *p* < .001, *d* = 0.38 |
| State Anxiety | *M* = 2.49, *SD* = 3.71 | *t*(364) = 12.83, *p* < .001, *d* = 0.67 |

**Study 1: Planned Contrast Results**

**Supplementary Table 5**

*Mean Differences, Standard Errors, and Inferential Statistics for Positive and Negative Affect and State Anxiety Change Scores, and Intrusion Frequency Scores and Intrusion Characteristic Ratings*

|  | **Mean Difference** | **SE** | ***t*** | ***p*** |
| --- | --- | --- | --- | --- |
| **Positive Affect** |  |  |  |  |
| Unmodified vs. Greyscale | -0.71 | 0.71 | -0.99 | .323 |
| Unmodified vs. Blur | -0.63 | 0.72 | -0.88 | .378 |
| Greyscale vs. Blur | -0.07 | 0.72 | -0.10 | .918 |
| **Negative Affect** |  |  |  |  |
| Unmodified vs. Greyscale | 0.38 | 0.70 | 0.54 | .587 |
| Unmodified vs. Blur | 0.54 | 0.71 | 0.76 | .445 |
| Greyscale vs. Blur | -0.16 | 0.71 | -0.22 | .822 |
| ***“Nervous” Item*** |  |  |  |  |
| Unmodified vs. Greyscale | 0.03 | 0.12 | 0.24 | .811 |
| Unmodified vs. Blur | -0.02 | 0.12 | -0.16 | .871 |
| Greyscale vs. Blur | 0.05 | 0.12 | 0.40 | .688 |
| ***“Jittery” Item*** |  |  |  |  |
| Unmodified vs. Greyscale | 0.06 | 0.11 | 0.51 | .613 |
| Unmodified vs. Blur | 0.05 | 0.11 | 0.46 | .646 |
| Greyscale vs. Blur | 0.00 | 0.11 | 0.04 | .965 |
| **State Anxiety** |  |  |  |  |
| Unmodified vs. Greyscale | 0.28 | 0.50 | 0.56 | .599 |
| Unmodified vs. Blur | 0.14 | 0.50 | 0.27 | .784 |
| Greyscale vs. Blur | 0.14 | 0.50 | 0.28 | .778 |
|  | **Mean Difference** | **SE** | ***t*** | ***p*** |
| **Intrusion Frequency** |  |  |  |  |
| Unmodified vs. Greyscale | 0.90 | 1.90 | 0.47 | .637 |
| Unmodified vs. Blur | 2.84 | 1.91 | 1.49 | .137 |
| Greyscale vs. Blur | -1.95 | 1.90 | 1.02 | .307 |
| **Intrusion Intrusiveness** |  |  |  |  |
| Unmodified vs. Greyscale | 0.12 | 0.27 | 0.46 | .644 |
| Unmodified vs. Blur | 0.17 | 0.28 | 0.60 | .550 |
| Greyscale vs. Blur | -0.04 | 0.27 | -0.16 | .875 |
| **Intrusion Distress** |  |  |  |  |
| Unmodified vs. Greyscale | -0.29 | 0.30 | -0.98 | .327 |
| Unmodified vs. Blur | -0.17 | 0.31 | -0.55 | .586 |
| Greyscale vs. Blur | -0.13 | 0.30 | -0.42 | .675 |
| **Intrusion Vividness** |  |  |  |  |
| Unmodified vs. Greyscale | -0.17 | 0.24 | -0.71 | .481 |
| Unmodified vs. Blur | -0.20 | 0.25 | -0.81 | .419 |
| Greyscale vs. Blur | 0.03 | 0.24 | 0.13 | .895 |
| **Intrusion Unwantedness** |  |  |  |  |
| Unmodified vs. Greyscale | 0.05 | 0.24 | 0.20 | .840 |
| Unmodified vs. Blur | 0.26 | 0.24 | 1.08 | .280 |
| Greyscale vs. Blur | -0.22 | 0.23 | -0.92 | .358 |
| **Intrusion Emotional Intensity** |  |  |  |  |
| Unmodified vs. Greyscale | -0.14 | 0.29 | -0.51 | .613 |
| Unmodified vs. Blur | 0.04 | 0.30 | 0.15 | .881 |
| Greyscale vs. Blur | -0.19 | 0.28 | -0.66 | .507 |
|  | **Mean Difference** | **SE** | ***t*** | ***p*** |
| **Intrusion Valence** |  |  |  |  |
| Unmodified vs. Greyscale | -0.08 | 0.20 | -0.38 | .706 |
| Unmodified vs. Blur | -0.02 | 0.21 | -0.08 | .933 |
| Greyscale vs. Blur | -0.06 | 0.20 | -0.29 | .772 |

**Study 2: PCL-5 Results**

**Supplementary Table 6**

*Pearson Correlations Between PCL-5 Scores and Main Dependent Variables*

| **Variable** | ***r*** |  | ***p*** |
| --- | --- | --- | --- |
| Positive Affect | -.16 | ** | .002 |
| Negative Affect | .07 |  | .182 |
| State Anxiety | -.02 |  | .735 |
| Intrusion Frequency | .12 | * | .019 |
| Intrusion intrusiveness | .20 | *** | <.001 |
| Intrusion distress | .26 | *** | <.001 |
| Intrusion vividness | .16 | ** | .006 |
| Intrusion unwantedness | -.01 |  | .883 |
| Intrusion emotional intensity | .28 | *** | <.001 |
| Intrusion valence | .03 |  | .570 |

*Note*. * *p* < .05, ** *p* < .01, *** *p* < .001

**Comparison Between Conditions**

Although not pre-registered, we checked whether participants who chose modified images scored higher on the PCL-5 than participants who chose unmodified images. There was no difference in PCL-5 scores between participants who chose unmodified images (*M* = 19.99, *SD* = 17.68) and participants who chose modified images (*M* = 18.89, *SD* = 16.99), *t*(363) = 0.14, *p* = .582, *d* = -0.06 (95% CI [-0.29, 0.16]), BF₁₀ = 0.15 (moderate evidence for H₀).

**Study 2: Self-Location Question Results**

As in Experiment 1, we used the self-location questions to gauge whether the image modification strategies made participants feel more distant from the images. Similar to our findings in Experiment 1, participants who chose unmodified images (*M* = 2.04, *SD* = 1.16) and participants who chose modified images (*M* = 2.06, *SD* = 1.08), felt similarly immersed in the images, *t*(363) = 0.14, *p* = .886, *d* = 0.02 (95% CI [-0.21, 0.24]), BF₁₀ = 0.13 (moderate evidence for H₀). These results suggest that *choosing* to view modified versus unmodified images made no difference to how immersed participants felt in the environment of the images (which, as in Experiment 1, was not very much).
